# Clinical, Laboratory and Histological Features of Dipeptidyl Peptidase-4 Inhibitor Related Noninflammatory Bullous Pemphigoid

**DOI:** 10.3390/jcm10091916

**Published:** 2021-04-28

**Authors:** Ágnes Kinyó, Anita Hanyecz, Zsuzsanna Lengyel, Dalma Várszegi, Péter Oláh, Csaba Gyömörei, Endre Kálmán, Tímea Berki, Rolland Gyulai

**Affiliations:** 1 Department of Dermatology, Venereology and Oncodermatology, University of Pécs Medical School Clinical Centre, H7632 Pécs, Hungary; hanyecz.anita@pte.hu (A.H.); lengyel.zsuzsanna@pte.hu (Z.L.); varszegi.dalma@pte.hu (D.V.); olah.peter@pte.hu (P.O.); gyulai.rolland@pte.hu (R.G.); 2 Department of Pathology, University of Pécs Medical School Clinical Centre, H7632 Pécs, Hungary; gyomorei.csaba@pte.hu (C.G.); kalman.endre@pte.hu (E.K.); 3 Department of Immunology and Biotechnology, University of Pécs Medical School Clinical Centre, H7632 Pécs, Hungary; berki.timea@pte.hu

**Keywords:** bullous pemphigoid, dipeptidyl peptidase-4 inhibitor, DPP4, eosinophil, gliptin

## Abstract

Bullous pemphigoid (BP) is an autoimmune blistering disease of elderly patients that has shown increasing incidence in the last decades. Higher prevalence of BP may be due to more frequent use of provoking agents, such as antidiabetic dipeptidyl peptidase-4 inhibitor (DPP4i) drugs. Our aim was to assess DPP4i-induced bullous pemphigoid among our BP patients and characterize the clinical, laboratory and histological features of this drug-induced disease form. In our patient cohort, out of 127 BP patients (79 females (62.2%), 48 males (37.7%)), 14 (9 females and 5 males) were treated with DPP4i at the time of BP diagnosis. The Bullous Pemphigoid Disease Area Index (BPDAI) urticaria/erythema score was significantly lower, and the BPDAI damage score was significantly higher in DPP4i-BP patients compared to the nonDPP4i group. Both the mean absolute eosinophil number and the mean periblister eosinophil number was significantly lower in DPP4i-BP patients than in nonDPP4i cases (317.7 ± 0.204 vs. 894.0 ± 1.171 cells/μL, *p* < 0.0001; 6.75 ± 1.72 vs. 19.09 ± 3.1, *p* = 0.0012, respectively). Our results provide further evidence that DPP4i-associated BP differs significantly from classical BP, and presents with less distributed skin symptoms, mild erythema, normal or slightly elevated peripheral eosinophil count, and lower titers of BP180 autoantibodies. To our knowledge, this is the first case series of DPP4i-related BP with a non-inflammatory phenotype in European patients.

## 1. Introduction

Bullous pemphigoid (BP) is an autoimmune blistering disease of elderly patients that has shown increasing incidence in the last decades [1,2,3,4,5,6,7,8]. Higher prevalence of BP may be due to the increasing mean age of the population, more frequent use of provoking drugs, such as loop diuretics, penicillin or PD-1/PD-L1 inhibitors, increased incidence of associated diseases (e.g., neurological diseases or hematologic malignancies), and recognition of atypical types of the disease [3,4,5,6,7,8]. However, some studies showed a higher prevalence of diabetes mellitus and a higher number of antidiabetic dipeptidyl peptidase 4 inhibitor (DPP4i) taking patients among BP patients [9,10,11,12,13,14,15,16,17,18,19]. An increasing number of studies showed association of DPP4i and bullous pemphigoid, but the exact mechanism of this association remains unclear [9,10,11,12,13,14,15,16,17,18,19,20,21,22,23,24]. As shown by several recent studies, DPP4i-related BP is associated with an atypical form, characterized by erosions without erythema, decreased eosinophil infiltration around the blisters and negative serological results to NC16A domain of BP180 [25,26,27,28,29]. This noninflammatory phenotype of the bullous disease was present in higher rates in patients taking DPP4i for several months, however, noninflammatory BP can also be found in nonDPP4i related cases, but in a significantly lower number [30,31].

The aim of our study was to analyze the clinical, laboratory and histological presentations of BP in our patients, and compare the clinical presentation of the DPP4i taking patients to the nonDPP4i-associated BP cases.

## 2. Materials and Methods

### 2.1. Patients

Patients diagnosed with BP between 1 January 2007 and 31 December 2017 at the Department of Dermatology, Venereology and Oncodermatology, University of Pécs, Hungary were enrolled into this retrospective study. The diagnosis of BP was based on the characteristic clinical features, the typical histopathological findings, and at least one of the following immunological features: (1) linear deposits of IgG and/or C3 along the basement membrane by direct immunofluorescence (DIF); (2) circulating autoantibodies detected by standard indirect immunofluorescent technique (IIF); (3) the presence of circulating IgG antibodies against BP180 using enzyme-linked immunosorbent assay (ELISA) [32].

### 2.2. ELISA

Samples for ELISA were only collected from 2015 onwards, and analysis was performed in 34 samples of 32 patients: 24 nonDPP4i and 6 DPP4i patients at disease onset, only 2 nonDPP4i patients in complete remission, and 2 nonDPP4i patients both at disease onset and in complete remission. IgG antibodies binding to BP180 and BP230 proteins were evaluated using commercial kits (Euroimmun, Lubeck, Germany). The ELISA kit for BP180 was specific for the NC16A domain.

### 2.3. Evaluation of Clinical Characteristics of BP

The clinical features of the BP patients were evaluated by two dermatologists experienced in BP management, based on the patients’ actual clinical status or retrospectively, based on photo documentation. The Bullous Pemphigoid Disease Area Index (BPDAI) was calculated in 59 patients (46 nonDPP4i and 13 DPP4i-BP) [33]. We have differentiated the patients as inflammatory and noninflammatory forms of BP based on the clinical manifestation of the disease. Classical or inflammatory forms of BP were defined by extended skin blistering with large bulla and urticarial erythema around the blisters, while the clinical characteristics of atypical or noninflammatory forms, were less extended skin lesions with smaller blisters and the absence of surrounding erythema in the periblister area.

### 2.4. Peripheral Eosinophilia

Serum eosinophil count was measured at first presentation before any therapeutic procedures. Eosinophilia was defined as ≥500 cells/μL absolute eosinophil count in peripheral blood, and it was further classified into three severity groups: mild (500 to 1500 cells/μL), moderate (1500 to 5000 cells/μL), or severe (≥5000 cells/μL) [31].

### 2.5. Periblister Eosinophilic Infiltration

To calculate the number of eosinophils in the skin tissue, biopsy specimens were collected from 45 BP patients (32 nonDPP4i and 13 DPP4i-BP). Skin samples were stained with hematoxylin-eosin, and the number of eosinophils were expressed as the mean value of the counted numbers in five random grids per section under ×400 magnification in the periblister area.

### 2.6. Statistical Analysis

Group means were compared using Student’s *t*-test with Welch’s correction in the case of unequal group variances. Pearson’s correlation coefficient was used to measure between-group correlations. Association of immunofluorescence results with DPP4i status was assessed using Fisher’s exact test. Statistical analyses were carried out using GraphPad Prism version 5 (GraphPad Software, La Jolla CA, San Diego, CA, USA) and Microsoft Excel.

This study was approved by the ethical committee of University of Pécs (7841-PTE 2019) and was performed according to the principles of the Declaration of Helsinki.

## 3. Results

### 3.1. Demographic Data of the Study Patients

One hundred and twenty-seven patients (79 females (62.2%), 48 males (37.7%)) with BP were enrolled in our study. The prevalence of type 2 diabetes mellitus (DM2) was 25.2% (32/127) at the time of diagnosis in our BP patients. Fourteen of the 32 DM2 patients were treated with DPP4i inhibitor at the time of the diagnosis of BP (9 females and 5 males). Of these, 12 patients were treated with vildagliptin (in 8 cases in combination with metformin), and 2 patients were treated with linagliptin (1 of them in combination with metformin). The onset of BP was several months after the introduction of DPP4i (range: 11–38 months). The mean age of patients was 75 years in nonDPP4i patients (range: 39–97 years), and 71 years in DPP4i patients (range: 49–92 years), there was no significant difference between the two groups (Table 1).

### 3.2. Clinical Characteristics

**Figure 1:**
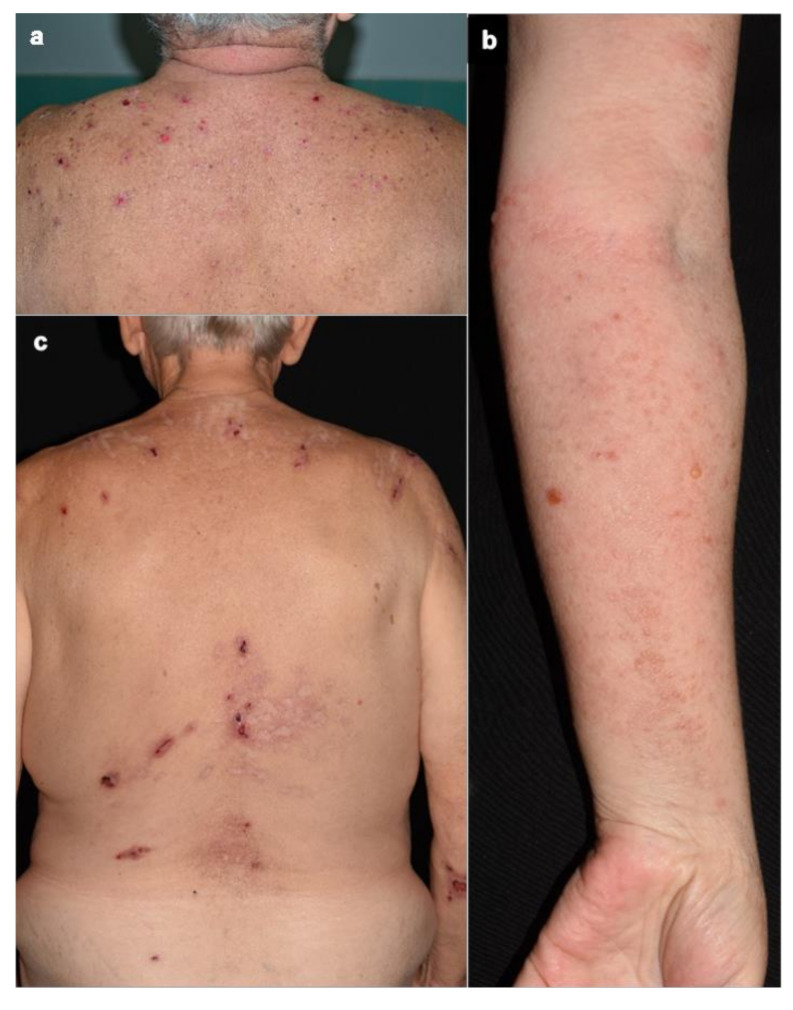
(**a**) Mild skin involvement localized to the upper part of the trunk with small, round erosions without erythema in a DPP4i-BP patient. (**b**) Small, herpetiform blisters with mild erythema on the forearm in a DPP4i patient. (**c**) Similar clinical lesions with hypo-, and hyperpigmentation and linear scars.

Clinical evaluation of BP characteristics and severity was performed in 59 patients (46 nonDPP4i and 13 DPP4i). The classical, inflammatory phenotype of BP, with tense bulla, prominent erythema and more disseminated distribution was present in 31 of 46 nonDPP4i-BP cases (67.4%), while in 15 patients (32.6%) we observed noninflammatory or other atypical BP forms. In 10 out of 14 DPP4i-BP patients (71.4%) the clinical phenotype was noninflammatory: mild extension, more prominent distribution of the lesions on the upper part of the trunk and extremities (Figure 1a,c), smaller blisters and erosions without erythema or urticarial lesions. In the remaining 4 patients (28.6%), disseminated bullous lesions were detected. Two of them had smaller, herpes-like blisters with surrounding scant erythema (Figure 1b), the other two had larger bullae, but the perilesional scar formation was so prominent that it was difficult to distinguish it from erythema. The presence of a noninflammatory or atypical form of BP was significantly higher in the DPP4i patients than in nonDPP4i-BP group (71.4% vs. 32.6%, respectively; *p* = 0.014).

The mean BPDAI erosions/blisters (BPDAI E/B) values were not significantly different between the DPP4i and nonDPP4i groups (22.9 vs. 24.8, respectively; *p* = 0.66). On the other hand, the mean BPDAI urticaria/erythema (BPDAI U/E) values were significantly lower in DPP4i-BP patients compared to the nonDPP4i group (6.8 vs. 16.5, respectively; *p* = 0.012) (Table 1). Clinically, damage including postinflammatory hyperpigmentation and scarring or erythema from resolving lesions was more frequent in DPP4i-BP patients. Thus, BPDAI damage values were significantly higher compared to the nonDPP4i group (2.5 vs. 0.9, respectively; *p* = 0.027) (Table 1). Oral mucosal involvement was present in 13/46 (28.2%) in nonDPP4i and 3/14 (21.4%) in DPP4i patients; there was no significant difference between the two groups in mucosal BPDAI (0.5 vs. 1; *p* = 0.6) (Table 1).

### 3.3. Eosinophilia in DPP4i and nonDPP4i-BP Patients

**Figure 2:**
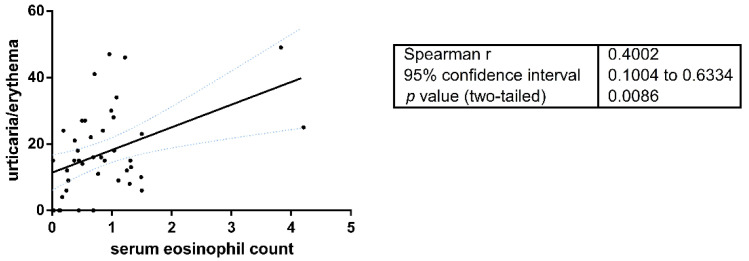
Significant correlation between circulating eosinophil count and BPDAI U/E values, the strength of the associations is expressed as Spearman’s correlation coefficient (r).

The serum eosinophil count was assessed at first presentation of the disease before any therapeutic procedure in 115 cases (14 DPP4i and 101 nonDPP4i-BP). The mean absolute eosinophil number in DPP4i-BP patients (n = 14) was significantly lower than in nonDPP4i cases (n = 101) (317.7 ± 0.204 vs. 894.0 ± 1.171, respectively; *p* < 0.0001) (Table 1). Fifty-one (50.5%) of 101 nonDPP4i patients had eosinophilia (≥500 cells/μL): 37 (36.6%) mild, 12 (11.8%) moderate, and 2 (1.9%) the severe form. Only 2 of the 14 (14.3%) DPP4i-BP patients had elevated eosinophil counts, both had mild eosinophilia. The peripheral eosinophil count proved to be in significant positive correlation with the urticaria/erythema scores of the BPDAI index (Spearman’s r = 0.4002; *p* = 0.0086) (Figure 2).

### 3.4. Periblister Tissue Eosinophilia

**Figure 3:**
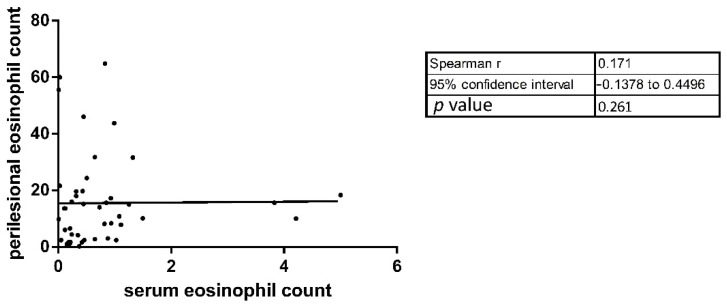
The correlation between serum and periblister eosinophil count was not significant.

**Figure 4:**
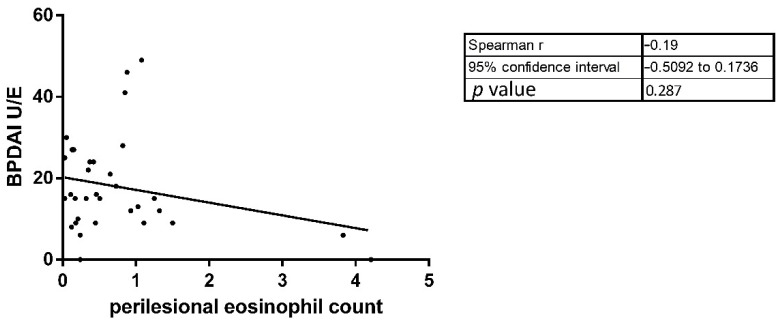
The correlation between the periblister eosinophil count and the BPDAI U/E value was also not significant.

To characterize the histopathological differences between DPP4i and nonDPP4i patients, we determined the number of eosinophils infiltrating into the dermis of periblister skin in 45 BP patients (13 DPP4i and 32 nonDPP4i-BP). The mean periblister tissue eosinophil number in DPP4i patients was significantly lower compared to the nonDPP4i cases (6.75 vs. 19.09; respectively; *p* = 0.0012) (Table 1). We observed a non-significant positive correlation (both *p* = 0.17) between the serum eosinophil number and the periblister eosinophil count, as well as the periblister eosinophil count and the BPDAI U/E values (Figure 3 and Figure 4).

### 3.5. Immunopathological Findings

Direct immunofluorescence results were available in 124 cases (110 nonDPP4i- and 14 DPP4i-BP). These showed C3 positivity in 106/110 (96.4%) in nonDPP4i vs. 11/14 (78.6%) in DPP4i patients; IgG positivity in 101/110 (91.8%) nonDPP4i vs. 13/14 (92.9%) DPP4i and IgA positivity in 10/110 (9.1%) vs. 1/14 (7.1%) DPP4i patients (Table 1). In DPP4i patients, C3 positivity was significantly lower (*p* = 0.031), but IgG and IgA positivity were statistically not different from nonDPP4i patients. In nonDPP4i patients, indirect IF was performed in 38 cases, 12 of these (31.6%) were positive, while all 6 tested DPP4i patients proved negative.

### 3.6. ELISA Results in nonDPP4i and DPP4i Patients

**Figure 5:**
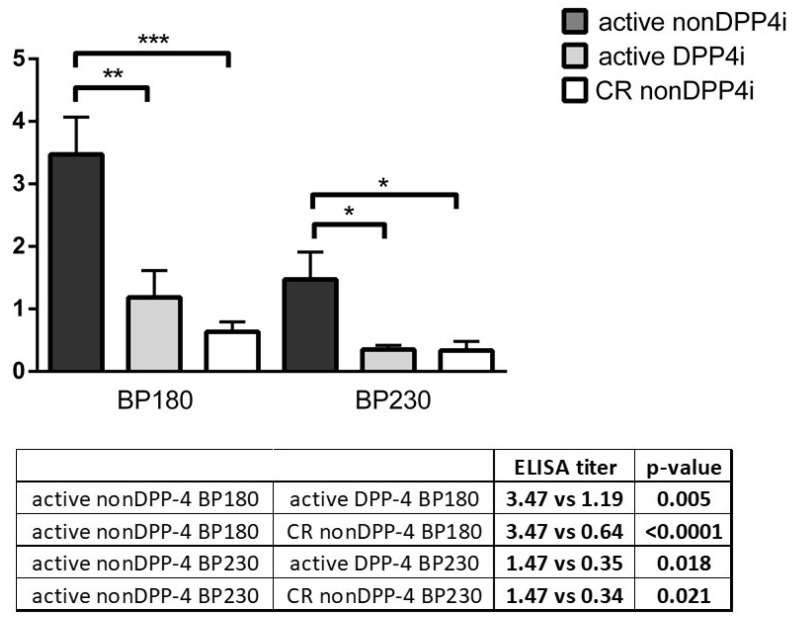
BP180 and BP230 ELISA titers of untreated DPP4i (active DPP4i), untreated nonDPP4i BP (active nonDPP4i) and treated nonDPP4i with complete response (CR nonDPP4i) BP patients; (* *p* < 0.05; ** *p* ≤ 0.01; *** *p* ≤ 0.001).

ELISA analysis was performed on 36 occasions in 30 patients (24 nonDPP4i and 6 DPP4i patients) (Figure 5). At initial diagnosis 17/24 nonDPP4i patients showed positivity for BP180 (70.8%), and 7 were positive for BP230 (29.1%). All of them showed markedly elevated titers for both BP180 (average titer value: 3.47) and BP230 (average titer value: 1.47). NonDPP4i patients in complete remission (CR) (n = 4) were negative for both BP180 (average titer value: 0.64) and BP230 (average titer value: 0.34). At initial diagnosis BP180 was positive in 3 (50%) of 6 DPP4i patients, and average titers were significantly lower than in active nonDPP4i patients (1.19 vs. 3.47; *p* = 0.005). Two of these three positive patients presented with noninflammatory phenotypes of BP. The third patient had an atypical clinical phenotype with maculopapular exanthemas on the trunk and small blisters on the extremities. None of the DPP4i patients showed positivity for BP230.

### 3.7. Patients’ Outcomes in DPP4i Patients

NonDPP4i patients were treated by conventional BP treatment according to the guidelines, with good therapeutic responses. In cases with limited extent we applied topical corticosteroid treatment; in mild or moderate cases, systemic corticosteroid or azathioprine alone, and in severe cases, systemic corticosteroid together with azathioprine, or methotrexate and/or diamino-diphenylsulphone. We had only one recalcitrant case who needed intravenous immunoglobulin treatment (Supplementary Table S1). In DPP4i patients, DPP4i was discontinued in only 3 of 14 patients because at the time of the diagnosis, the provoking role of the DPP4i was not recognized, or the patient was not willing to discontinue the drug. Two patients were treated with topical corticosteroid, eight patients received only oral systemic corticosteroid, and four patients needed adjuvant immunosuppressive drug in addition to the corticosteroid treatment (azathioprine in three cases, diamino-diphenylsulphone in one case). The clinical outcome with the relevant therapy in DPP4i patients was similar to nonDPP4i patients, irrespective of the continuation or the withdrawal of DPP4i.

## 4. Discussion

During the last two decades both the incidence of BP and the number of BP patients with diabetes taking DPP4i has been increasing [1,2,3,4,5,6,7,8,9,10,11,12,13,14,15,16,17,18,19,20,21,22,23,24,25,26,27,28,29,30,31]. Although DPP4 inhibitors are often used in combination with metformin, the association between BP and DPP4i proved to be independent of the use of metformin [23,34]. Among the currently available DPP4 inhibitors, vildagliptin was most frequently associated with BP; however, more recently linagliptin has also been strongly linked to BP induction [13,14,16,18,19,23,35,36,37,38]. Compared to previous studies, in our patient population vildagliptin had the strongest association with BP, and a weaker association was also observed with linagliptin, despite the fact that sitagliptin is the most common prescribed DPP4i in Hungary (56.9%), followed by vildagliptin (31.8%) and litagliptin (9%) [39]. These data have also underlined the role of vildagliptin and linagliptin in the occurrence of BP. Although sitagliptin, saxagliptin and alogliptin are also approved and prescribed in diabetes in Hungary, these drugs were not detected in our study population. Two recent European multicenter investigations showed that DPP4i-induced BP is more common in male patients [13,16]. Similar to Varpoulama et al., our findings showed that women were more likely than men to develop DPP4i-associated BP [35].

The clinical and immunological characterization of DPP4i-associated BP has been the focus of several recent studies [23,24,25,26,27,28,29,30,31]. First, Izumi et al. [25] showed a noninflammatory form of DPP4i-associated BP in 7 patients, with limited distribution of smaller blisters, scant erythema and sparse periblister eosinophilic infiltration. Subsequent reports confirmed that this noninflammatory phenotype is more frequently associated with DPP4i-BP [26,28]. This unique, noninflammatory BP phenotype, however, has only been observed to date in Japanese DPP4i-patients [40]. In addition to the noninflammatory presentation, mucosal involvement was also more frequent and more severe (as shown by higher mucosal BPDAI scores) in DPP4i patients, compared to nonDPP4i patients [27,41]. Furthermore, in DPP4i-associated BP, lower peripheral and perilesional eosinophil count values were reported, although these differences have been somewhat inconsistent, and statistically not always significant [23,27,42,43]. Nevertheless, an association between eosinophils and BP has long been established, and previous studies also demonstrated a correlation between the eosinophil count and the severity of BP and the BPDAI [44,45].

In this study we have differentiated a noninflammatory form of BP induced by DPP4 inhibitor gliptins. These patients showed a mild extension of the disease; lesions predominantly involved the upper extremities and the upper part of the trunk. Individual lesions presented as solitary erosions without surrounding erythema and blisters were often small, herpetiform vesicles. The lack of erythema around the lesions in our DPP4i patients was in accordance with significantly decreased eosinophilic infiltration in the periblister area. Lower BPDAI U/E values, normal or slightly elevated serum eosinophil levels, and sparse periblister eosinophilic infiltration were detectable in DPP4i-induced BP patients. BPDAI U/E values were also in correlation with the serum eosinophil count. These findings further emphasize the non-inflammatory presentations of DPP4i inhibitor-related BP. On the other hand, BPDAI damage score values were significantly higher in DPP4i BP patients. This finding is not consistent with the noninflammatory manifestations of the disease in this group, but the higher prevalence of hypo- and hyperpigmentation, and scar formation were more likely related to the underlying diabetes and consequent worse wound healing in DPP4i patients. We did not detect any significant difference in mucosal involvement between nonDPP4i and DPP4i-BP patients; however, the presence of mucosal involvement was relatively high in our nonDPP4i patients compared to the earlier published data [40]. Interestingly, the effectivity of the therapy and the clinical outcome was independent of the withdrawal of the gliptins, and similar to the observation of Plaquevent et al. [24].

Previously it was found that patients with noninflammatory BP do not show reactivity against the immunodominant NC16A domain of BP180 but are positive to full-length BP180 and its ectodomain midportion with ELISA [25]. On the contrary, García-Díez et al. [30] demonstrated that 6 of 8 DPP4i taking BP patients (four of them with noninflammatory phenotype, and four with mucosal involvement) were positive for the NC16A domain of BP180. Horikawa et al. [26] found that 7 of 12 patients had autoantibodies against the NC16A domain and the remaining 5 patients were positive for full-length BP180. BPDAI (U/E) was significantly lower in the anti-NC16A negative cases. Fania et al. [46] and Yoshiji et al. [31] also reported NC16A positive cases, but these patients presented with evident erythema and inflammation in contrast to the other noninflammatory phenotypes of DPP4i-associated cases. Mai et al. [28] and Takama et al. [47] reported noninflammatory DPP4i-induced BP patients whose sera reacted with the full-length BP180 and did not react to the NC16A domain initially, but became positive later during their disease course. García-Díez et al. [48] reported a similar case with inflammatory BP, whose initial negative ELISA results became positive months later. In our DPP4i patients, autoantibody titers were significantly lower than in nonDPP4i patients, consistent with the findings of Ständer et al. [49] and Ujiie et al. [50], but the small subset of the DPP4i patients limits the conclusions that may be drawn from this finding. However, some previous reports also showed comparable autoantibody titers in DPP4i-related and non-DPP4i-related BP patients [21,27,51]. Antibodies against the NC16A domain of BP180 were detected initially in three patients (two of them with noninflammatory BP). Interestingly, none of our DPP4i patients showed BP230 reactivity. Furthermore, in contrast with previously published results, indirect IF was negative in all our DPP4i patients, [23,30,52] and the proportion of C3 positivity was lower among DPP4i patients. This result supports the hypothesis that, although in most BP cases C3 can be detected along the basement membrane, dermal-epidermal separation may also develop in a complement-independent manner [53].

To date, the noninflammatory BP phenotype was mainly observed in Japanese DPP4i-patients, while European databases do not show significant differences between DPP4i and nonDPP4i BP patients [25,26,28,40]. In Japanese BP patients, a higher prevalence of HLA-DQB1*03:01 was found, and this allele positivity was more common in DPP4i-induced BP patients with noninflammatory symptoms [11,50]. In a Finnish study they have found association of HLA-DQB1*03:01 with BP, but did not detect a difference between DPP4i and nonDPP4i-associated cases based on the presence or absence of the allele, and did not find any noninflammatory phenotype due to the gliptin-intake [51]. In our BP patient cohort, we observed several DPP4i-associated cases with a predominantly noninflammatory form, lower peripherial and periblister tissue eosinophil count, and higher rate of hyperpigmentation and/or scarring. Interestingly, it has been shown previously that HLA-DQB1*03:01 may be involved in the presentation of immunodominant epitopes of BPAG2 to autoreactive T cells in BP [54]. While the exact pathomechanism of DPP4i-related BP has not been clearly understood, it is known that DPP4 (also known as CD26) is expressed on the cell surface of immune cells, including T cells [55,56]. Thus, DPP4 inhibitors, apart from their well-known antihyperglycemic effects, may affect immune functions, specifically T cell behavior [56,57]. Furthermore, it has been reported that inhibition of DPP4 blocks eosinophil migration to the skin in rats [58]. HLA allele frequencies were not investigated in our BP population, but it may be assumed that DQB1*03:01 may also be overrepresented in our Hungarian patients. In addition to its direct immune effects, DPP4 also has intrinsic enzyme activity that activates plasminogen, and increases plasmin levels [59,60]. Plasmin digests BP180, and cleavage of COL17 within the NC16A domain induces conformational changes and neoepitopes with increased antigenicity [59,60,61]. The inhibition of plasmin by DPP4 inhibitors may thus suppress or change the development of epitopes within the NC16A domain, which may be associated with noninflammatory BP [25,59,60].

## 5. Conclusions

Taken together, we have shown that in our cohort of patients, DPP4i-related BP is more likely to present with a noninflammatory BP phenotype, decreased peripheral and skin eosinophilia, significantly lower BP180 antibody titers, a lower proportion of C3 positivity, and negative indirect IF and BP230 antibodies, as compared to nonDPP4i-BP. Limitations of our study include the small sample subset of ELISA results in DPP4i patients and the retrospective design of the investigation.

## Figures and Tables

**Table 1:** Comparison of clinical, laboratory and histological findings in DPP4i-related and nonDPP4i BP patients.

		nonDPP4i	DPP4i	*p* Value
**Age *(y, mean)***		75.4	71.1	NS
**Sex Female**		70/113 (61.9%)	9/14 (64.2%)	NS
**Atypical form of BP**		15/46 (32.6%)	10/14 (71.4%)	0.014
**BPDAI** *(mean)*	E/B	24.8	22.9	0.66
	U/E	16.5	6.8	0.012
	Damage	0.9	2.5	0.027
	Mucosa	1.1	0.5	0.6
**Mucosal involvement**		24/113 (21.2%)	3/14 (21.4%)	NS
**Serum eosinophil count** *(cells/μL, mean)*		0.894 (n = 101)	0.317 (n = 14)	<0.0001
**Periblister eosinophil count** *(mean)*		19.09 (n = 32)	6.75 (n = 13)	0.0012
**DIF**	**C3**	106/110 (96.4%)	11/14 (78.6%)	0.031
	**IgG**	101/110 (91.8%)	13/14 (92.9%)	NS
	**IgA**	10/110 (9.2%)	1/14 (7.1%)	NS
	**IgM**	2/110 (1.8%)	0/14 (0%)	NS
**ELISA** *(mean)*	**BP180**	3.47	1.19	0.005
	**BP230**	1.47	0.35	0.018

BP: bullous pemphigoid; DPP4i-BP: dipeptidyl peptidase-4 induced bullous pemphigoid; nonDPP4i: idiopathic pemphigoid; BPDAI: Bullous Pemphigoid Disease Area Index; BPDAI E/B Bullous Pemphigoid Disease Area Index erosions/blisters; BPDAI U/E: Bullous Pemphigoid Disease Area Index urticaria/erythema.

## Data Availability

The data presented are available and can be provided from the corresponding author on request.

## Supplementary Materials

The following are available online at https://www.mdpi.com/article/10.3390/jcm10091916/s1, Supplementary Table S1: Comparison of patients’ outcomes in nonDPP4i and DPP4i patients.
