# Supplementary material for: Clinical, Laboratory and Histological Features of Dipeptidyl Peptidase-4 Inhibitor Related Noninflammatory Bullous Pemphigoid
Source: J Clin Med. 2021 Apr 28;10(9):1916. doi: 10.3390/jcm10091916 (PMC8125701; doi:10.3390/jcm10091916)
Supplement: Supplementary file 1 [file jcm-10-01916-s001.zip › jcm-1181265-supplementary.pdf]

Supplementary Table S1. Comparison of patients' outcome in nonDPP4i and DPP4i patients.

	NonDPP4i n=113	DPP4i n=14
Topical steroid	**8**	**2**
Systemic CTS	**46**	**8**
Systemic CTS + azathioprine	**46**	**3**
Systemic CTS + diamino-diphenylsulphon	**1**	**1**
Systemic corticosteroid + methotrexate	**5**	**0**
Azathioprine	**2**	**0**
Systemic CTS + azathioprine + diamino-diphenylsulphon	**1**	**0**
Systemic CTS + azathioprine + diamino-diphenylsulphon + IVIg	**1**	**0**
No information	**3**	**0**

CTS: corticosteroid; IVIg: intravenous immunoglobulin treatment
